# Genetic markers of ovarian follicle number and menopause in women of multiple ethnicities

**DOI:** 10.1007/s00439-012-1184-0

**Published:** 2012-06-13

**Authors:** Sonya M. Schuh-Huerta, Nicholas A. Johnson, Mitchell P. Rosen, Barbara Sternfeld, Marcelle I. Cedars, Renee A. Reijo Pera

**Affiliations:** 1 Department of Obstetrics and Gynecology, Institute for Stem Cell Biology and Regenerative Medicine, Center for Human Embryonic Stem Cell Research and Education, School of Medicine, Stanford University, 265 Campus Drive, Lorry I. Lokey Stem Cell Research Building Rm G1165, Stanford, CA 94305 USA; 2 Department of Statistics, Stanford University, Stanford, CA 94305 USA; 3 Department of Obstetrics, Gynecology and Reproductive Sciences, University of California San Francisco, San Francisco, CA 94115 USA; 4 Division of Research, Kaiser Permanente, Oakland, CA 94612 USA

## Abstract

Oocyte loss has a significant impact on fertility and somatic health. Yet, we know little about factors that impact this process. We sought to identify genetic variants associated with ovarian reserve (oocyte number as measured by antral follicle count, AFC). Based on recently published genome-wide scans that identified loci associated with age of menopause, we also sought to test our hypothesis that follicle number and menopausal age share underlying genetic associations. We analyzed menopause-related variants for association with follicle number in an independent population of approximately 450 reproductive-aged women of European and African ancestry; these women were assessed for AFC, anthropometric, clinical, and lifestyle factors. One SNP strongly associated with later menopausal age in Caucasian women (+1.07 ± 0.11 years) in previous work was also associated with higher follicle counts in Caucasians (+2.79 ± 1.67 follicles) in our study. This variant is within the *Minichromosome Maintenance Complex Component 8* (*MCM8*) gene, which we found was expressed within oocytes in follicles of the human ovary. In genome-wide scans of AFC, we also identified one marginally genome-wide and several nominally significant SNPs within several other genes associated with follicle number in both ethnic groups. Further, there were overlapping variants associated with multiple ovarian reserve markers (AFC, serum hormone levels, menopausal age). This study provides the first evidence for direct genetic associations underlying both follicle number and menopause and identifies novel candidate genes. Genetic variants associated with ovarian reserve may facilitate discovery of genetic markers to predict reproductive health and lifespan in women.

**Electronic supplementary material:**

The online version of this article (doi:10.1007/s00439-012-1184-0) contains supplementary material, which is available to authorized users.

## Introduction

Infertility is remarkably common in humans compared to other species and affects nearly 15 % of reproductive-aged couples (Hull et al. 1985; Menken and Larsen 1994; Speroff 2005). In some cases, especially in the male, infertility is linked to genetic defects in germ cell development that manifest as reduced quantity or quality of sperm (Hull et al. 1985; Reijo Pera et al. 1995; Xu et al. 2001). However, other than genetic loci identified in certain reproductive disorders and animal models, genes involved in human female fertility, in particular the formation and maintenance of oocytes, have yet to be discovered. Indeed, to date, no genome-wide association studies (GWASs) have directly investigated follicle (oocyte) number in women.

In the human female, the number of oocytes increases initially in fetal development and then declines, from an established pool, beginning in prenatal life. Early in development (~3 weeks post-conception), a small population of founder cells, the primordial germ cells (PGCs), migrates to the genital ridges and rapidly proliferates to about 5–7 million germ cells. These PGCs, now termed gonocytes, subsequently begin meiosis and differentiate within the developing ovaries (Larsen 1997). The meiotic oogonia, arrested in prophase I, become surrounded by supportive somatic (granulosa) cells to form the follicles. Through the process of atresia (cell death), oocyte and follicle loss results in about 1 million follicles within the ovaries at birth (Block 1953). Over the course of a woman’s lifetime oocytes may be recruited to resume meiosis (to mature and undergo ovulation), remain quiescent, or be lost through atresia, as is the case for the vast majority of oocytes. By the time of puberty approximately 500,000 follicles remain, declining to 10,000–50,000 by the late 30s; eventually, the follicle pool is exhausted and menopause ensues (Block 1952, 1953; Faddy et al. 1992; Hansen et al. 2008; Richardson et al. 1987; te Velde and Pearson 2002; te Velde et al. 1998).

The age-related decline in the oocyte pool and the associated increase in chromosomal aneuploidies and miscarriage rates have important implications for understanding human oocyte biology and fertility (Hunt and Hassold 2008). There is great variability in the quantity and quality of the oocyte pool, or *ovarian reserve*, among women. There is also wide variability in reproductive potential and the timing of reproductive events such as menarche (onset of the first menstrual period) and menopause, both of which likely have strong genetic and environmental influences (Cramer et al. 1995; de Bruin et al. 2001; Gosden et al. 2007; Snieder et al. 1998; te Velde and Pearson 2002). Family and twin studies indicate that menarche and menopause have high heritability (~50–80 %) and genetic components (de Bruin et al. 2001; Meyer et al. 1991; Murabito et al. 2005; Snieder et al. 1998). Furthermore, the process of ovarian aging and timing of reproductive events are important risk factors for various diseases including breast and ovarian cancer, cardiovascular disease, osteoporosis, and overall mortality (Cooper and Sandler 1998; Hartge 2009). However, due to the inaccessibility of early human development we know little of the genes and variants involved in oocyte development and establishment of the ovarian reserve. No studies have explored the relationship between follicle number, menopause, and associated genes. This is likely because there are few studies examining follicle number in normo-ovulatory women and it is nearly impossible to study follicle number and menopause in the same cohort of women—in all previous studies on menopause, data on follicle number were not available or would have likely been too low to measure.

An underlying objective of this work was to identify genetic markers of ovarian reserve that would enable more accurate long-range predictions about reproductive and somatic health. Here, we used a community-based cohort of reproductively normal women of European and African ancestry of the OVA (Ovarian Aging) Study to investigate genetic loci associated with the phenotypic traits of follicle number and menopause. We hypothesized that ovarian reserve and reproductive lifespan are closely associated and largely determined by multiple genetic factors that may impact early germ cell and follicle formation, or rate of follicle loss. We tested the newly identified variants associated with menopausal age in previous cohorts for association with antral follicle number (a stage of oocyte readily observed by ultrasound) and other ovarian reserve markers in this completely independent population of women. Additionally, we conducted a GWAS to determine whether specific variants may be associated with the ovarian reserve as assessed by antral follicle count, in these ethnic groups. Finally, the expression of identified candidate genes was examined in human ovarian tissue.

## Subjects and methods

### Study population

The study cohorts included 273 Caucasian women and 245 African American women, aged 25–45 years. All subjects were ovulatory with regular menstrual cycles between 22 and 35 days in length, and enrolled in the OVA Study (an ongoing, multi-ethnic, community-based study). Women with oligo- or an-ovulation, surgically diagnosed endometriosis, premature ovarian failure (POF), polycystic ovary syndrome (PCOS), hyperandrogenism, or a history of uterine or ovarian surgery were excluded. Women were also excluded if they had cysts, fibroids or other abnormalities that obscured AFC measurements. Ethnicity was by self-report but required both parents to be of the same, identified ethnicity. Of the 273 Caucasian women, 21 were excluded, and of the 245 African American women, 27 were excluded, due to anatomical or ovarian problems (Online Resource Table 1). For a detailed description of the OVA Study population and additional information on all methods see the Online Resource material. This work was approved by the Institutional Review Boards at Kaiser Permanente, University of California San Francisco, and Stanford University, and informed consent was obtained for all subjects.

### Phenotypes and covariates

Antral follicle counts were performed as previously described (Rosen et al. 2010b; Schuh-Huerta et al. 2012). Briefly, all subjects underwent transvaginal ultrasound assessment of ovarian volumes and AFC, performed on days 2–4 of their menstrual cycle. Blood samples were collected at the same time and used for all serum hormonal assays and genotyping as in previous work (Schuh-Huerta et al. 2012). Subjects completed a comprehensive questionnaire and physical exam to obtain detailed health, reproductive, anthropometric (body), lifestyle, and demographic information.

### Genotyping

Genotypic data for a total of 249 Caucasian and 203 African American women were obtained using the Genome-Wide Human SNP Array 6.0 (Affymetrix, Santa Clara, CA, USA). As previously published (Schuh-Huerta et al. 2012), genomic DNA was extracted and purified from white blood cells using the QIAamp DNA Blood Maxi Kit (Qiagen, Venlo, The Netherlands) according to the manufacturer’s instructions. The DNA ligation, hybridization, and microarray scanning steps were completed according to the manufacturer’s protocols. Briefly, 500 ng total genomic DNA was prepared at 50 ng/μl using the Nanodrop ND-1000 Spectrophotometer (Nanodrop Technologies, Wilmington, DE, USA), aliquoted, digested into fragments, ligated to adaptors, amplified by PCR, and purified using polystyrene beads. The amplified DNA was fragmented (<180 bp/fragment), labeled, hybridized to the microarray, stained, and washed using the Fluidics Station 450, and then scanned with the GeneChip Scanner 3000 7G (Affymetrix).

In the Caucasian group, after removal of one sample failing quality control (QC), two samples failing ethnicity validation, and one duplicate sample, of the final 245 samples that passed QC and filter criteria, the mean contrast QC was 1.93 ± 0.046, the QC call rate was 94.0 ± 0.2 %, and the overall sample call rate was 98.0 ± 0.07 %. In the African American group, after removal of one sample failing ethnicity validation, of the final 202 samples the mean contrast QC was 2.22 ± 0.035, the QC call rate was 97.0 ± 0.1 %, and the overall sample call rate was 99.4 ± 0.08 %. SNPs were genotyped using Genotyping Console (GTC) v.4.0 software (http://www.affymetrix.com). All SNP locations are referenced to the National Center for Biotechnology Information (NCBI) human genome build 37, February 2009, and dbSNP build 131, April 2010 (http://www.ncbi.nlm.nih.gov/projects/SNP). Gene names provided in all tables and text represent the HUGO gene symbols (http://www.genenames.org/).

### Population structure

A principal component analysis (PCA) using singular value decomposition was used for ethnicity validation and tests of population structure. Subjects of the HapMap CEU (Utah residents with N. and W. European ancestry from the CEPH collection) and YRI cohorts (Yoruban in Ibadan, Nigeria) (International HapMap Consortium 2005) (http://hapmap.ncbi.nlm.nih.gov/) were genotyped using the same genotyping platform, for comparison with the OVA cohorts, as described (Schuh-Huerta et al. 2012). PCA was performed using the statistical program R v.2.11.1 (http://www.r-project.org/) (Gentleman et al. 2008) and BEAGLE v3.0.2 (http://www.stat.aukland.ac.nz/~bbrowning/beagle/beagle) (Browning and Browning 2007; Browning 2006). Fraction of African ancestry was also determined and allele frequency spectra were compared between the Caucasian and CEU, and African American and CEU/YRI cohorts to analyze allele composition and population homogeneity.

### Immunofluorescence

To examine the expression of MCM8 and the established germ cell/oocyte marker *VASA* (*DEAD* (*Asp*-*Glu*-*Ala*-*Asp*) *Box Polypeptide 4, DDX4*) within the ovary, human ovarian tissue was freshly harvested and donated from a 37-year-old healthy Caucasian woman. She had no known ovarian dysfunction or other diseases. Human testicular tissue was also obtained from a 29-year-old healthy Caucasian male with no reported reproductive or somatic diseases. The tissues were fixed in 10 % neutral buffered formalin, embedded in paraffin, sectioned (at a thickness of 5–7 μm), and mounted on positively charged slides (Zyagen, San Diego, CA, USA). The sections were washed three times in xylene for 4 min each to promote dewaxing, followed by successive ethanol washes for rehydration: three times in 100 % ethanol for 4 min, two times in 95 % ethanol for 3 min, 90 % ethanol for 3 min, 85 % ethanol for 3 min, 80 % ethanol for 3 min, 70 % ethanol for 3 min, 50 % ethanol for 3 min, and dH_2_O for 3 min. Antigen retrieval was performed following dewaxing and rehydration, by incubating the sections 10 min in prewarmed citrate buffer (5 mM trisodium citrate, 4.4 mM HCl, pH 6.0) then boiling in citrate buffer in a rice cooker for 25–30 min. Slides were air-dried, traced with a PAP pen (Sigma-Aldrich, St. Louis, MO, USA), and rinsed three times with PBS (Sigma-Aldrich). Tissues were then permeabilized in 0.2 % Triton X-100 (Sigma-Aldrich) in PBS for 15 min at room temperature. Slides were washed three times in PBS and blocked to prevent non-specific binding in 4 % normal donkey serum (Jackson ImmunoResearch Labs, Inc., West Grove, PA, USA) in PBS overnight at 4 °C in a humid chamber. The slides were then incubated for 1 h at room temperature with the rabbit anti-human MCM8 primary antibody (SC99038; Santa Cruz Biotechnology Inc., Santa Cruz, CA, USA) at dilutions of 1:100 and 1:200, and the goat anti-human VASA antibody (AF2030; R&D Systems, Minneapolis, MN, USA) at 1:250, in PBS with 4 % donkey serum. As a negative control, adjacent sections were incubated with the rabbit and goat non-immune isotype IgGs at 1:500 (Invitrogen, Grand Island, NY, USA) in place of the primary antibody. As an additional biological negative control, mature human neurons derived and cultured in vitro were stained for MCM8 and VASA using the same antibodies and protocols. Slides were washed three times with PBS supplemented with 0.1 % Tween-20 (PBST) and primary signals were detected using the donkey anti-rabbit secondary antibody conjugated to Alexa Fluor-488 and the anti-goat secondary antibody conjugated to Alexa-Fluor-594 (Invitrogen) at 1:500 in PBS with 4 % donkey serum for 1 h at room temperature in the dark. Slides were washed three times with PBST, one time with PBS, stained with DAPI (1:5,000) for 1 h at 4 °C in the dark, mounted with coverslips, and imaged on a Zeiss LSM700 Meta Inverted laser scanning confocal microscope and a Leica DMI 6000B Inverted fluorescence microscope. The same microscopy settings including pinhole, laser power, exposure time, and gain were used for all sections. All fluorescence images were processed in ImageJ (NIH).

### Statistical analyses

#### Phenotypic and covariate analysis

Descriptive statistics were calculated for AFC and all demographic, anthropometric, hormonal, reproductive, and lifestyle variables. The relationship between each variable and AFC was analyzed in linear regression analyses. Phenotypes and covariates were also independently compared between Caucasian and African American women, using the Student’s *t* test or Welch 2-Sample *t* test within R v.2.11.1 (Gentleman et al. 2008). Covariates that were significantly associated with follicle number were controlled for in downstream GWA analyses.

#### SNP association analysis

In candidate SNP studies, we searched for and analyzed all previously reported top SNPs associated with menopause that had corresponding markers on our microarrays. A total of four SNPs significant at the genome-wide level (*P* < 5.0 × 10^−8^) and replicated in previous work were tested for association with follicle count in both the Caucasian and African American cohorts. Final *P* values were corrected for the number of tests and considered statistically significant at a level of <0.05. Effect sizes in terms of direction (increase/decrease in AFC or menopausal age) and magnitude (total difference in the mean number of follicles or menopausal age in years) for the effect allele/genotype were compared for all variants tested. As approximately one antral follicle is lost each year of female age and presumably women with fewer antral follicles enter menopause sooner, quantitative comparisons can be made between the differences in the mean AFC and mean menopausal age for all effect alleles.

Both candidate SNP and GWA analyses were performed using the Fisher’s exact test for single-marker (SNP) and multiple-marker (haplotype) association. The correlation coefficients and effect sizes (the difference in the mean number of follicles ± SEM from the regression fit) were then determined by regression analyses for a given allele or genotype. Final *P* values were computed and analyzed at both the chromosome- and genome-wide levels, corrected by permutation testing and Bonferroni correction, due to our sample size/power constraints. For detailed power calculations refer to Online Resource, “Subjects and methods.” Custom-made script and algorithms were written and employed within *R* to mediate streamlined data analyses.

## Results

### Characteristics of study population

**Fig. 1:**
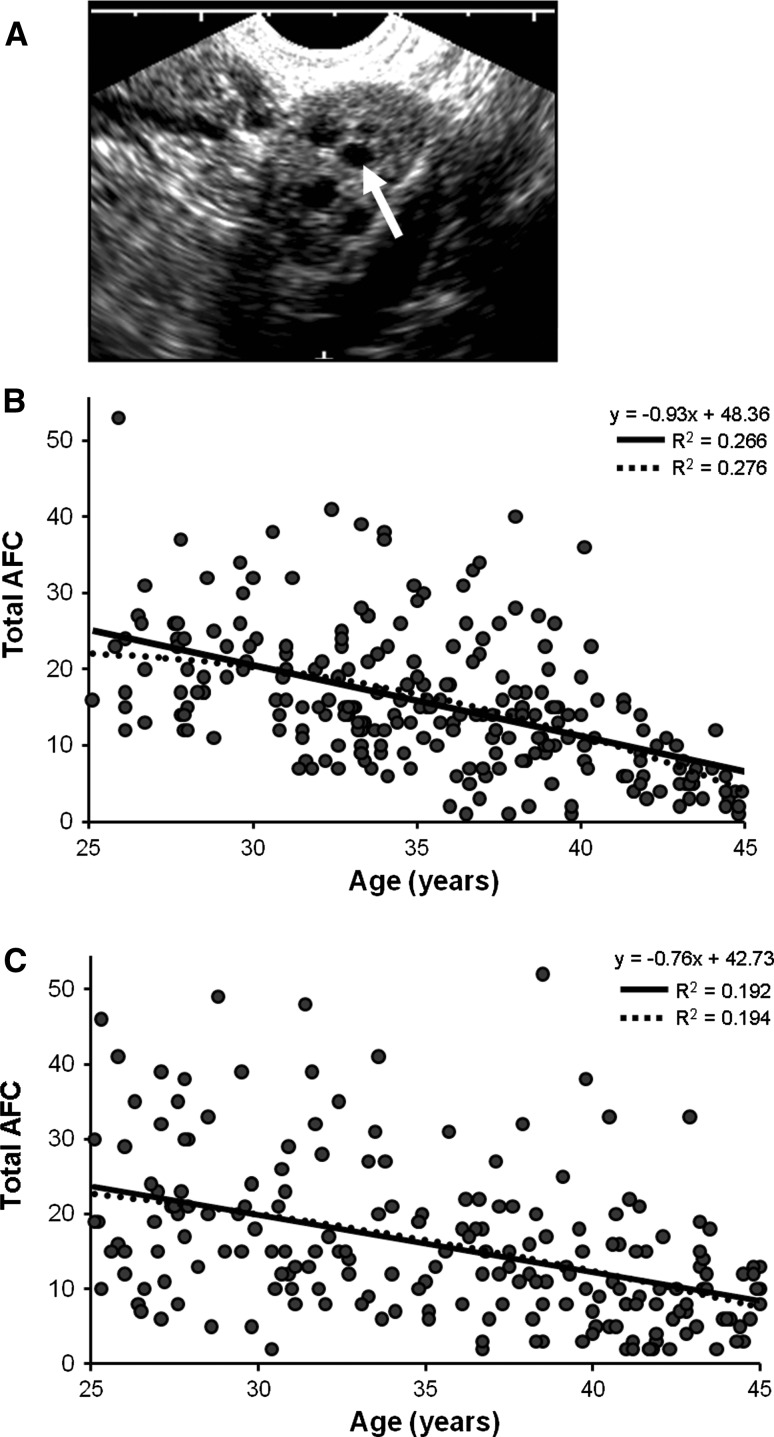
Measurements of antral follicle count in women. **A** Transvaginal ultrasound image of the left ovary of one woman showing antral follicles ranging in size (*arrow* indicates one large antral follicle of 8–10 mm). Total antral follicle counts obtained from **B** Caucasian and **C** African American women of the entire study population indicate that AFC decreases with age, but is highly variable between women. The data were fit with the power model (*dashed line*) and linear regression model (*solid line*). The corresponding correlation coefficients (*R* ^2^) and linear equations are shown

As shown in Fig. 1 and Online Resource Figure 1, AFC measurements, blood samples, and questionnaires were collected from 273 Caucasian women and 245 African American women. After applying quality control filters and removal of women with anatomical or ovarian abnormalities, a total of 245 women of genetically inferred European ancestry and 202 women of genetically inferred African ancestry were included in the final analysis and genotyped for 909,622 SNPs. The relevant demographics of the study population used in genetic association analyses are described in Table 1. The average age was 35.4 ± 0.3 and 35.6 ± 0.4 (mean ± SEM) years for the Caucasian and African American cohorts, respectively, and the ages were normally distributed with a slight left-ward shift. AFCs ranged from 1 to 53 with a mean of 15.4 ± 0.6 in the Caucasian cohort, and from 2 to 52 with a mean of 15.6 ± 0.7 in the African American cohort. As reported in our recent work (Rosen et al. 2010b), in the women genotyped in this study AFC was negatively associated with age. The relationship between AFC and age in both Caucasian and African American women was best fit by simple linear regression and power [AFC = *A* + *B* × (age)^*C*^] models. As shown in Fig. 1B, C, AFC declined with female age, with a gradual acceleration of follicle loss beginning around age 40. This pattern was similar in both Caucasian and African American women. The average decline in AFC was about 0.93 follicles/year in Caucasians and 0.76 follicles/year in African Americans, although there was great variability among both groups. There were no statistically significant differences between the mean values or regressions of AFC verses age between the two racial groups. Further, there was no association between percentage of African ancestry and AFC as determined from PCA and regression analyses, as described below. However, there was a trend for higher follicle numbers and a greater distribution of follicle counts above 35, as well as greater variability in the African American women (Fig. 1C).

**Table 1:** Demographics of study population and variables included in association analysis of antral follicle count

	Caucasians			African Americans		
	Mean ± SEM^a^	*n*	*R* ^2b^	Mean ± SEM^a^	*n*	*R* ^2b^
Age	35.4 ± 0.3	245	**−0.27**	35.6 ± 0.4	202	**−0.20**
AFC	15.4 ± 0.6	245	1.00	15.6 ± 0.7	202	1.00
Height (cm)	166.4 ± 0.4 (5′6″)*	243	0.00020	164.3 ± 0.5 (5′5″)*	202	0.015
Weight (kg)	67.7 ± 1.0 (149 lbs)*	243	0.0034	87.0 ± 1.7 (192 lbs)*	202	−0.0048
BMI	24.4 ± 0.4*	243	0.0046	32.1 ± 0.6*	201	−0.012
Age at Menarche	12.8 ± 0.09*	243	−0.022	12.1 ± 0.1*	200	−2 × 10^−6^
Cycle length (days)	29.1 ± 0.1	243	0.0014	29.6 ± 0.2	200	0.0077
Parity (≥1 birth)	36 %	243		52 %	200	

Asterisks indicate significant differences between Caucasian and African American women (*P* < 0.001)

^a^Values are shown as mean ± SEM or percent of the cohort

^b^Correlation coefficient is based on regression with AFC. Bold values indicate highly significant correlation (*P* < 0.001)

### Population structure

In PCA tests of population stratification there was a close clustering of the Caucasian cohort with the HapMap CEU cohort and the African American cohort with the HapMap YRI cohort (Online Resource Figure 2). Two Caucasian samples were identified as non-Caucasian, and clustered with the African American cohort, while one African American sample was identified as Caucasian and clustered with the Caucasian and CEU populations (Online Resource Figure 2; Table 1). These individuals self-identified as the race they were originally categorized as; outliers unlikely resulting from sampling error. The Caucasian cohort displayed an allele frequency spectrum nearly identical to the CEU population, while the African American cohort displayed an allele frequency spectrum representing a combination of 80 % YRI and 20 % CEU alleles which is exactly the allele spectra of individuals of African ancestry living in America (Online Resource Figure 2B). Moreover, both the Caucasian and CEU, and the African American and YRI/CEU cohorts, respectively, showed highly similar genotype frequencies and minor allele frequencies, validating the homogeneity and ethnic identity of our cohorts (Online Resource Figure 2B). In regression analyses using PC1 and PC2 scores (fraction of African ancestry), when controlling for female age, AFC was not associated with African ancestry (*P* = 0.57 and 0.81).

### Genetic association analysis

To examine the association between follicle number and reproductive lifespan we analyzed the relationship between menopause-related SNPs identified in previous work, with follicle number and other ovarian reserve markers, in this cohort of women. This strategy aimed to provide both validation and a test of the prediction that these two traits share underlying genetic loci. We reasoned that the large, well-characterized post-menopausal populations of these previous studies might provide some validation of our findings and with our data, critical insight on the genetic links between follicle number and menopause. We predicted an approximate 1:1 ratio between the difference in menopausal age:follicle number for these variants, with effects going in the same directions for both traits if they are functionally related—and this is indeed the case. A total of four genome-wide significant SNPs from three previous publications (He et al. 2009; Murray et al. 2010; Stolk et al. 2009) were tested for association with follicle count using the Fisher’s exact test in both the Caucasian (*n* = 223) and the African American (*n* = 202) cohorts. As shown in Table 2, SNP rs16991615 at loci 20p12.3, strongly associated with a later menopausal age of +1.07 ± 0.11 years per copy of the minor allele (He et al. 2009), was significantly associated with higher follicle counts of +2.79 ± 1.67 follicles for the corresponding genotype in the Caucasian women (*P* = 0.018). This SNP was within the *MCM8* (*Minichromosome Maintenance Complex Component 8*) gene and was associated with a delayed age of menopause at the genome-wide level in approximately 9,000 women of the Nurse’s Health and Women’s Genome Health Studies (He et al. 2009), 3,000 women of the Rotterdam Baseline and Twins UK Studies (Stolk et al. 2009), and replicated in approximately 2,000 women of the Breakthrough Generations and ReproGen Studies (Murray et al. 2010).

**Table 2:** Associations between known menopause-related SNPs and follicle number in Caucasian women of OVA Study

SNP^a^	Gene	Cytoband	Alleles^b^	MAF	Menopause GWAS genotype effect (years)^c^	OVA study genotype effect (follicles)^d^	*P* value
rs16991615	*MCM8*	20p12.3	A/G	0.07	+1.07 ± 0.11	+2.79 ± 1.67	**0.0045** (**0.018**)
rs4806660	*TMEM150B*	19q13.42	G/A	0.37	−0.41 ± 0.030	−1.77 ± 1.32	0.43 (1)
rs691141	*HK3*	5q35.2	A/G	0.48	+0.36 ± 0.052	+3.16 ± 1.32	0.34 (1)
rs2153157	*SYCP2L*	6p24.2	A/G	0.45	+0.29 ± 0.052	+2.21 ± 1.21	0.57 (1)

Genes: *MCM8* minichromosome maintenance complex component 8, *TMEM150B* transmembrane protein 150B, *HK3* hexokinase 3 (White cell), *SYCP2L* synaptonemal complex protein 2-like

Bold value indicates significance of *P* < 0.05

*P* values are based on the Fisher’s exact test for SNP association with antral follicle count; values in parentheses denote final corrected *P* values

^a^SNPs at each locus are those published for association with menopausal age, rather than those with the strongest signal for follicle count

^b^Minor/major allele

^c^The difference in mean (±SEM) age at menopause in years per copy of the SNP minor allele as previously reported

^d^The difference in mean (±SEM) number of follicles for the corresponding genotype calculated from the regression analysis

Interestingly, although none of the other menopausal SNPs that reached genome-wide significance in previous studies was independently significant at the 0.05 level in our study, *all* of them exhibited regression coefficients that were similar in magnitude and direction. There were highly similar allele/genotype effects on both menopausal age and follicle number, with a concordance between later menopausal age and higher follicle counts, and earlier menopausal age and lower follicle counts (Table 2). As AFC declines by ~1 follicle per year of female age, this is consistent with the observed associations between AFC and menopausal age for all variants tested. These results, and the ability to discover multiple variants associated with both menopausal age and follicle number, are likely limited by our sample size. Nevertheless, these results demonstrate for the first time a plausible functional link between menopausal genetic variants and oocyte number in women of European ancestry.

To further analyze the potential association of age at menopause with ovarian reserve, we examined the variants linked with menopausal age for association with several well-known markers of ovarian reserve, including AFC, anti-Mullerian horomone (AMH), and follicle-stimulating hormone (FSH). We found that the four menopausal SNPs in addition to SNPs in the 50-kb region surrounding the lead SNP were not independently associated with follicle number in the African American cohort (Online Resource Table 2). And in several cases the directions and magnitudes of effects were inconsistent between previous studies in Europeans and current studies in African Americans. This is not entirely unexpected due to the differences in ancestral background, LD structure, haplotypes, and allelic heterogeneity across different racial/ethnic groups. Because the minor allele frequency (MAF) for the *MCM8* SNP is so much lower in the African American group (i.e. 0.01) compared to the Caucasian group, these results should be interpreted with caution. However, variants in the regions immediately flanking the top menopausal SNP, rs16991615, had significantly low *P* values associated with AMH and FSH, in both ethnic groups. Seven to 11 variants had *P* values of 1.23 × 10^−2^ to 1.38 × 10^−3^ and were within a flanking region of 35–89 SNPs. These variants were also 2.5–10 times more likely than expected, based on *P* value distributions. Notably, the majority of these variants were linked with AMH, FSH, and/or AFC, and were replicated in both Caucasian and African American women. Several of these overlapping variants (rs237416, rs6053673, rs11700084, rs4618122, rs4407314, rs11908294, and rs6053911) were within the *MCM8*, *C20orf196* (*Chromosome 20 Open Reading Frame 196*), and *FERMT1* (*Fermitin Family Member 1*) genes. Together, these results provide greater validation and evidence for genetic associations underlying ovarian reserve and menopausal age in women of multiple ethnicities.

**Fig. 2:**
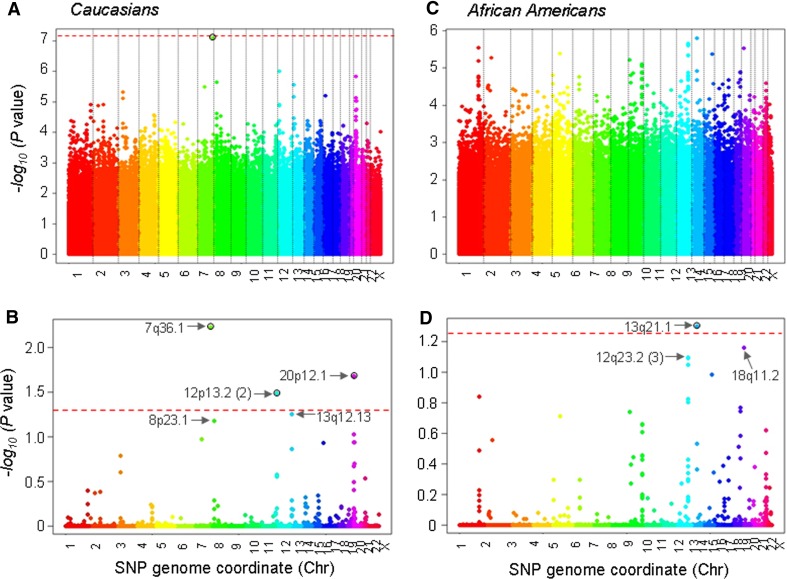
Summary of GWA results for follicle number. Manhattan plots of the **A**, **C** uncorrected and **B**, **D** corrected *P* values of the Fisher’s exact test in Caucasian (**A**, **B**) and African American (**C**, **D**) women. Each point represents a SNP from the single SNP and haplotype tests of association across the genome remaining after quality control and SNP filtering. The *red dashed lines* indicate *P* values of 5.0 × 10^−8^ (uncorrected) and 0.05 (chromosome corrected), significance level

In genome-wide analyses, we examined associations with AFC in the independent Caucasian and African American cohorts. SNPs with MAF of <1 %, missing call rate (MCR) of >5 %, significant deviation from Hardy–Weinberg Equilibrium (HWE) and high discordance rates were excluded, leaving 677,261 SNPs in the Caucasian cohort and 738,185 SNPs in the African American cohort that were independently tested for association with AFC. Figure 2 shows the Manhattan plots depicting association results across the genome in the Caucasian (Fig. 2A, B) and African American (Fig. 2C, D) cohorts. 

**Fig. 3:**
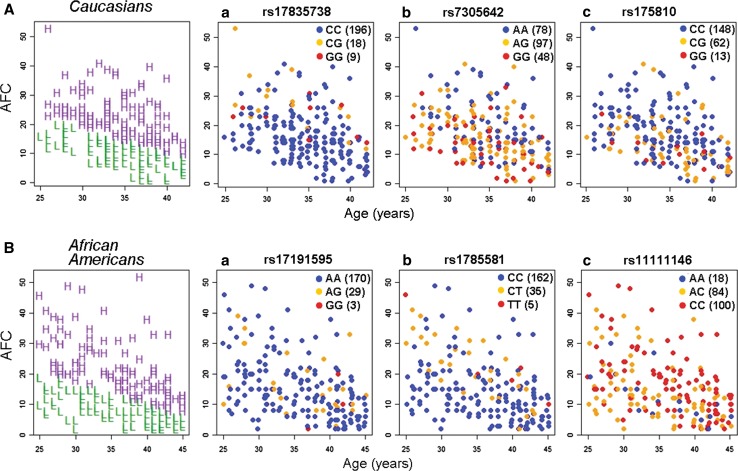
Phenotypes and genotypes of SNPs associated with AFC. **A**, **B** Shown in the *left panels* are *scatter plots* of the phenotypes of high (*H*) and low (*L*) AFC separated by robust regression. **a**–**c** Also shown is the distribution of genotypes among the women with respect to age and AFC for the top SNPs in the **A** Caucasian and **B** African American populations. The three genotypes are designated by *blue*, *orange* and *red points* as indicated in the legends (*parentheses* indicate the number of women with each genotype)

In the Caucasians, the top five genetic loci were on chromosome 7, 12 (2 SNPs), 20, 13, and 8 (Fig. 2). One SNP, rs17835738, located at 7q36.1, just upstream of the *Leucine Rich Repeat Containing 61* (*LRRC61*) gene and within the *ARP3 Actin*-*Related Protein 3 Homolog C* (*ACTR3C*) gene, was strongly associated with AFC at the chromosome-wide level (*P* = 0.006; MAF = 0.08) and was the only variant marginally associated at the genome-wide level (*P* = 7.9 × 10^−8^; Table 3; assuming a genome-wide threshold of *P* < 5.0 × 10^−8^). The other five SNPs were nominally associated with AFC (*P* = 9.76 × 10^−7^ to 2.77 × 10^−6^; MAFs = 0.08–0.43). As shown in Fig. 3, graphical representation of SNP genotypes with their corresponding phenotypes allows visualization of the association with low or high follicle counts. The most significant variant, rs17835738, was associated with mean increases of 6.9 ± 2.0 and 8.2 ± 2.3 follicles for the GG and GC genotypes, respectively, compared to 0.3 ± 0.5 follicles for the CC genotype (Fig. 3; Table 3). Variants rs7305642 and rs2417903 at 12p13.2 are located immediately downstream of the *Killer Cell Lectin*-*Like Receptor Subfamily A, Pseudogene 1* (*KLRAP1*). Variant rs7305642 was associated with an increase of 4.7 ± 1.0 follicles and a decrease of 2.2 ± 1.0 follicles for the AA and GG genotypes, respectively. Variant rs2417903 was associated with a decrease of 2.2 ± 1.0 and an increase of 4.7 ± 1.0 follicles for the AA and TT genotypes, respectively. Variant rs175810 at 20p12.1 is in an intronic region of the *MACRO Domain Containing 2* (*MACROD2*) gene and was associated with a decrease of 3.1 ± 1.3 follicles and an increase of 2.5 ± 0.7 follicles for the GG and CC genotypes, respectively. Of the last two nominal variants, rs4769524 at 13q12.13, is located upstream of the *G Protein*-*Coupled Receptor 12* (*GPR12*) gene and rs1382566 at 8p23.1, is located within an intron of the *B Lymphoid Tyrosine Kinase* (*BLK*) gene.

**Table 3:** Variants associated with follicle number

SNP	Chr	Gene	Position	Cytoband	Alleles^a^	MAF	AA effect^b^	AB effect^b^	BB effect^b^	*P* value
Caucasians										
rs17835738	7	*LRRC61*	149992098	q36.1	G/C	0.08	6.89 ± 1.95	8.17 ± 2.31	0.29 ± 0.54	**7.90** **×** **10** ^**−8**^
rs7305642	12	*KLRAP1*	10705382	p13.2	G/A	0.44	−2.24 ± 0.99	0.10 ± 0.72	4.67 ± 0.98	9.76 × 10^−7^
rs2417903	12	*KLRAP1*	10704691	p13.2	A/T	0.43	−2.24 ± 0.98	0.10 ± 0.72	4.67 ± 0.98	9.76 × 10^−7^
rs175810	20	*MACROD2*	15827930	p12.1	G/C	0.20	−3.14 ± 1.29	−1.09 ± 0.90	2.53 ± 0.67	1.48 × 10^−6^
rs4769524	13	*GPR12*	27377221	q12.13	A/G	0.08	n/a	−0.56 ± 0.96	1.51 ± 0.61	2.78 × 10^−6^
rs1382566	8	*BLK*	11384841	p23.1	C/G	0.15	6.34 ± 3.91	2.69 ± 1.10	0.51 ± 0.62	2.30 × 10^−6^
African Americans										
rs17191595	13	*DIAPH3*	58777413	q21.1	G/A	0.08	0.36 ± 6.14	3.26 ± 1.74	1.76 ± 0.72	1.55 × 10^−6^
rs1785581	18	*TAF4B*	23997267	q11.2	T/C	0.11	10.70 ± 3.57	6.76 ± 1.32	0.69 ± 0.73	2.98 × 10^−6^
rs11111146	12	*CCDC53*	102452264	q23.2	A/C	0.30	−1.73 ± 1.34	−0.25 ± 0.96	4.40 ± 0.98	2.21 × 10^−6^

Genes: *LRRC61* leucine rich repeat containing 61, *KLRAP1* killer cell lectin-like receptor subfamily A, pseudogene 1, *MACROD2* MACRO domain containing 2, *GPR12* g protein-coupled receptor 12, *BLK* b lymphoid tyrosine kinase, *DIAPH3* diaphanous homolog 3 (Drosophila), *TAF4B* TAF4b RNA polymerase II, TATA box binding protein (TBP)-associated factor, *CCDC53* coiled-coil domain containing 53

*P* values are based on the Fisher’s exact test for SNP association with antral follicle count. Bold values indicate genome-wide significance

^a^Minor/major allele

^b^The difference in mean (±SEM) number of follicles for a given genotype. “A” denotes the minor allele and “B” denotes the major allele

In replication studies, the top variants associated with AFC were examined in the African American population as an independent replication cohort. The top SNP rs17835738 was not significant at a *P* < 0.05 level. Although, this may be due to differences in minor allele frequencies (0.08 vs. 0.01 in the Caucasians and African Americans, respectively). However, in careful analyses of the SNPs flanking this variant and that of the other top six hits associated with AFC, it was found that for several of these there were clusters of nearby SNPs (12–150 SNPs away) that had significantly low *P* values of 1.9 × 10^−2^ to 2.1 × 10^−3^. Based on observed *P* value distributions there was a two- to sixfold greater number of these variants than expected by chance. This provides some validation of these genetic loci in the African American cohort.

In the African American cohort, the top three loci linked with AFC were localized to chromosome 13, 18, and 12 (Fig. 2C, D). Variant rs17191595 at 13q21.1, downstream of the *Protocadherin 17* (*PCDH17*) and *Diaphanous Homolog 3* *(Drosophila)* (*DIAPH3*) genes, was associated with AFC at the chromosome-wide level (*P* = 0.049; MAF = 0.08), but not at the genome-wide level (Table 3). This SNP was associated with mean increases of 1.8 ± 0.7 and 3.3 ± 1.7 follicles for the AA and AG genotypes, respectively (Fig. 3; Table 3). Of the other two nominal variants, rs1785581 at 18q11.2 is immediately downstream of the *TAF4b RNA Polymerase II, TATA Box Binding Protein* (*TBP*)-*Associated Factor* (*TAF4B*) gene, and was associated with increases of 10.7 ± 3.6 and 6.8 ± 1.3 follicles for the TT and CT genotypes, respectively. Rs11111146 at 12q23.2 is located in an intron of the *Coiled*-*Coil Domain Containing 53* (*CCDC53*) gene and was associated with a decrease of 1.7 ± 1.3 and an increase of 4.4 ± 1.0 follicles for the AA and CC genotypes, respectively (Fig. 3; Table 3).

A comparison of loci associated with follicle number and menopausal age indicates that several variants were clustered in specific genomic regions on chromosomes 20, 13, and 12 (Online Resource Figure 3). The variants on chromosome 20 including one associated with follicle number in Caucasians (rs175810), one within *MCM8* associated with both follicle number and menopause (rs16991615), and several nearby associated variants, were in a genomic region that comprises long stretches of highly associated SNPs. Moreover, we found that many of the identified variants associated with AFC were also associated with AMH (Online Resource Table 3; Fig. 5). Of the top 16 variants associated with AFC, seven of these were significantly associated with AMH levels, and again, localized to chromosomes 12, 13 and 20.

### MCM8 protein is expressed in human ovary

**Fig. 4:**
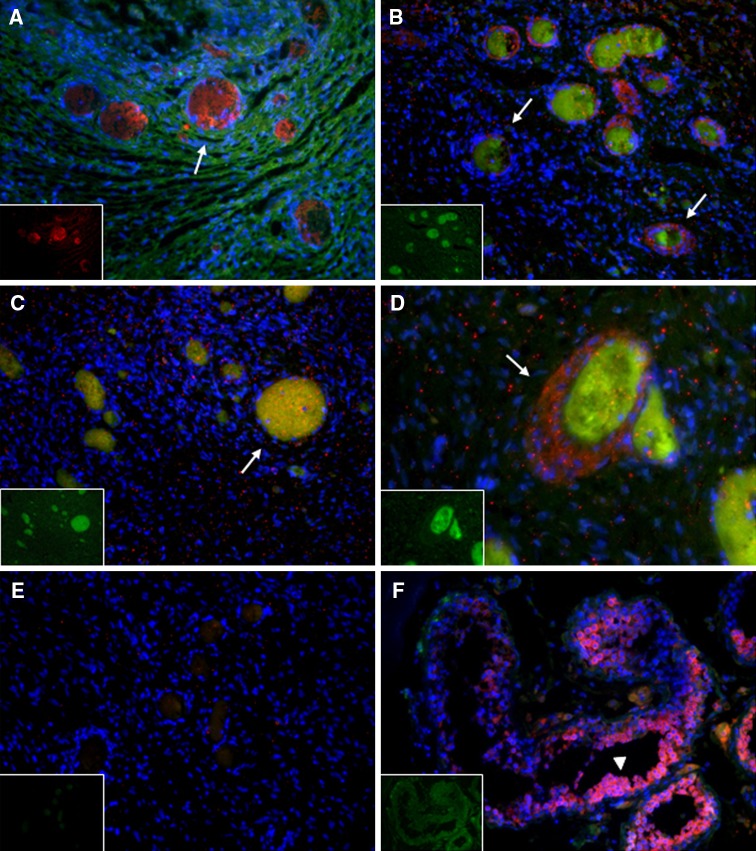
MCM8 is expressed in human ovarian follicles. **A**–**D** Immunodetection of the germ cell marker VASA (*red*) and MCM8 (*green*) in human ovarian tissue, showing colocalization in human oocytes (*yellow*-*orange*; *arrows* indicate primary and secondary follicles; several primordial and primary follicles also shown). **E** Lack of immunostaining in ovarian tissue incubated with rabbit and goat non-immune isotype IgGs in place of the primary antibodies. **F** VASA and MCM8 expression in adult human testis (*arrowhead* indicates developing sperm with high expression of VASA). For all images DNA was stained with DAPI (*blue*) (×200; *insets* show specific VASA or MCM8 staining)

The presence of MCM8 in human ovary was determined by immunohistochemistry and confocal and fluorescence microscopy. Slide-mounted ovarian tissue sections from a 37-year-old woman showed a positive signal for the anti-MCM8 antibody, visualized with the Alexa Fluor-488 secondary antibody (Fig. 4A–D). As shown in Fig. 4, MCM8 colocalized with the germ cell-specific marker VASA and was present within the oocytes of primordial, primary, and secondary follicles. No specific staining was observed with the rabbit and goat non-immune isotype IgG controls using the same conditions and secondary antibodies (negative control; Fig. 4E). MCM8 was detected at low levels in peritubular cells, but not in developing sperm (spermatocytes, spermatids, and sperm) in the seminiferous tubules of the human testis (Fig. 4F). Further, MCM8 was not detected in terminally differentiated human neurons (data not shown).

### Phenotypes and covariates

Anthropometric and reproductive measurements were all within the normal range and comparable to that of previous data (Table 1). See Online Resources for a detailed description of the population phenotypes and covariates. In brief, the African American cohort had significantly shorter height (*P* = 0.00035), greater weight (*P* = 2.54 × 10^−21^), higher waist-hip (*P* = 1.02 × 10^−18^) and waist-height (*P* = 2.47 × 10^−31^) ratios, and significantly greater BMI (*P* = 1.58 × 10^−27^). Interestingly, in the analysis of fraction of African ancestry, when controlling for female age BMI was significantly associated with amount of African ancestry (*P* = 0.014 and 0.012). Individuals with greater African ancestry had significantly higher BMIs. However, neither African ancestry nor BMI was associated with follicle number (*P* = 0.57 and 0.81, respectively). A significant proportion of the women had demonstrated fertility with 36 and 52 % of the Caucasian and African American women, respectively, having a parity of one or more children, and the remainder having no indications of reproductive disorders or infertility.

## Discussion

We evaluated genetic associations with ovarian reserve and validated variants associated with menopause, in an independent cohort of women directly assessed for follicle number. Our results indicate that there are underlying variants/genes associated with both ovarian reserve and menopause, providing more evidence for the connection between these two traits. Importantly, we found one of these genes, *MCM8,* is expressed within follicles of the adult human ovary and is therefore an excellent candidate gene for future studies of human oocyte development and ovarian function. Our work carries population-level genome-wide findings to the cellular level and provides likely functional evidence for a role of *MCM8* in ovarian biology. We also identified several additional SNPs associated with follicle number in Caucasian and African American women, with genetic loci that were validated in the different ethnic cohorts. These findings provide further evidence for a genetic basis of the reproductive lifespan and prospective markers and genes for future studies and clinical diagnostics.

Because of the close association between ovarian reserve and female fertility, as well as disease risk, studies have investigated the ability of various non-invasive markers to serve as proxies for ovarian reserve and predictors of reproductive potential. The ability to predict oocyte loss and reproductive lifespan would enable improved reproductive planning and health care decisions.

**Fig. 5:**
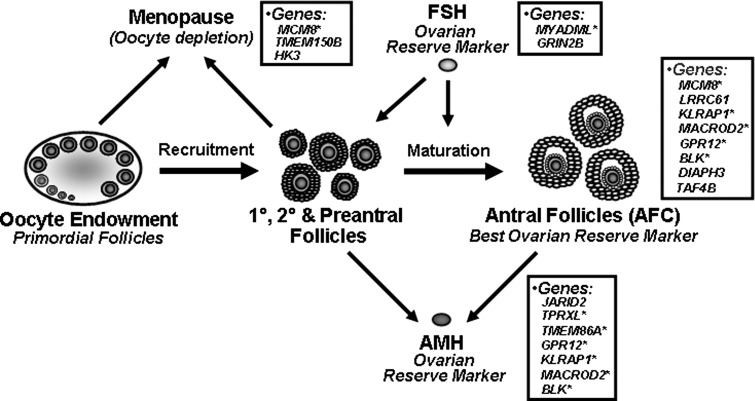
Depiction of the development, maturation, and depletion of the human female oocyte endowment and associated ovarian reserve markers and candidate genes. Current work identifies AFC as a good clinical marker and FSH and AMH as two good hormonal serum markers of ovarian reserve. Shown in *boxes* are the identified genes containing variants associated with AFC, FSH, AMH and menopause. These represent novel candidate genes for future studies of human ovarian function (*asterisks* indicate genes associated with two or more ovarian reserve markers)

Our recent work examined genetic variants and environmental factors associated with hormone markers of ovarian reserve, including FSH and AMH (Schuh-Huerta et al. 2012). As previously reported, there were several variants associated with AMH or FSH, that were also associated with follicle number; four out of the nine top variants were validated for association with AFC. These variants and genes linked with FSH and AMH, including those also significantly associated with AFC are shown in Fig. 5 (asterisks indicate overlapping genes). Because of the close relationships between these serum hormone levels, especially AMH, and total number of antral follicles, this prompted a closer examination of specific genetic factors associated with follicle number. The number of preantral and antral follicles to a large extent drives serum AMH levels, and therefore genetic associations with AMH levels might in large part be driven by follicle number. And this is indeed the case. Here, we report that many of the identified variants associated with AFC are also associated with AMH (Online Resource Table 3; Fig. 5). Of the top 16 variants associated with AFC, seven of these are significantly associated with AMH levels, encompassing the *GPR12*, *KLRAP1*, *MACROD2*, and *BLK* genes. Therefore, there are both overlapping and independent genetic markers associated with follicle number and other accurate markers of ovarian reserve.

AFC is considered one of the most reliable non-invasive methods for determining the ovarian reserve. Antral follicles are small (millimeters in diameter), fluid-filled structures that contain the developing oocytes and are visible by ultrasound. The assumption that the number of antral follicles is proportional to the total oocyte pool remaining in the ovary is supported by histologic data (Hansen et al. 2008; Morris et al. 2002) and has led investigators to compare the relationship between AFC and age (Broekmans et al. 2004; Faddy et al. 1992; Rosen et al. 2010b; Scheffer et al. 1999). In mathematical models that examined reproductive events in non-contracepting populations, AFC was predictive of age at natural fertility loss (~10 years prior to menopause) and age at menopause (Broekmans et al. 2004). AFC is also lower in infertile women (Rosen et al. 2011). Our prior work detected great variability in antral follicle count among Caucasian women (Rosen et al. 2010b) and a lowered AFC associated with an earlier maternal age of menopause (Rosen et al. 2010a), suggesting a strong genetic component to ovarian reserve. Indeed, maternal age of menopause is a predictor for AFC such that women whose mothers entered menopause earlier, have lower AFCs at any given age (Rosen et al. 2010a). However, no previous studies have identified genetic links with follicle number or between follicle number and menopause.

Candidate gene and linkage association studies, mostly in the context of reproductive disorders such as POF (menopause before age 40), have identified a limited number of genes and variants that may be associated with the follicle pool and ovarian aging (Bretherick et al. 2008; Fassnacht et al. 2006; Gromoll and Simoni 2005; Kevenaar et al. 2007; Simpson 2008; Suzumori et al. 2007; Tung et al. 2006; Yoon et al. 2010). The X chromosome has a significant role in ovarian function. Monosomy X results in women with Turner syndrome who have accelerated follicle loss, degeneration of ovaries, and generally lack functional oocytes, rendering them infertile (Fassnacht et al. 2006; Turner 1972). Various forms of POF, in about 1–4 % of women, are often familial and linked with genetic and structural abnormalities of the X chromosome (Fassnacht et al. 2006). However, in many POF cases, the underlying genetic variants or mutations are unknown. Putative roles of other female fertility genes have come from transgenic mouse models and include various proteins and transcription factors of oogenesis/folliculogenesis (Fassnacht et al. 2006; Knight and Glister 2006; Suzumori et al. 2007). While POF is rare, the genetic variants responsible for the normal variation in ovarian reserve (oocyte number) in the general population have not been identified.

Recently, a few large-scale GWASs have discovered SNPs associated with age of menarche (Elks et al. 2010; He et al. 2009; Ong et al. 2009; Sulem et al. 2009) and age of menopause (He et al. 2009; Murray et al. 2010; Stolk et al. 2009). In these large cohorts of post-menopausal European women, several SNPs led to increases or decreases of about 3 months to 1 year in age at menopause, further implicating genetic factors in the timing of follicle loss. Menopausal age is an indirect, retrospective marker of follicle loss; direct measurements of follicle number were not performed in these studies. Direct studies of follicle number and menopausal age in the same women have not been possible. Moreover, most previous work has focused on infertile populations, small observational studies, or post-menopausal women. Much data are lacking on genetics and reproductive parameters in women of reproductive age, without ovarian disorders, and not seeking infertility treatments. There is also little data on women of non-European ancestry. Therefore, our community-based cohort of reproductive-aged women of multiple ethnicities is an ideal population to investigate genetic loci associated with follicle number, and in combination with previous cohorts, menopause. GWA studies in this population can also determine whether additional specific variants may be associated with ovarian reserve markers and various fertility and somatic disorders in different ethnic groups.

We found that antral follicle number declined with female age, showing a similar pattern in both Caucasian and African American women, although with a trend for slightly greater follicle counts, greater variability, and a slower extrapolated rate of follicle loss in the African Americans compared to Caucasians (0.76 vs. 0.93 follicles/year, respectively). To our knowledge there are no other publications on follicle number in fertile African American women, so these results cannot be compared to any previously examined cohorts. It has been reported that African American women may have an earlier age at menopause, 6–12 months sooner than Caucasians (Bromberger et al. 1997), while other studies have found no difference in menopausal age between the two racial groups (Gold et al. 2001). Our results on follicle number indicate that this African American population does not appear to have a reduced ovarian reserve or increased risk for entering menopause earlier than the Caucasian population.

There were many differences in anthropometric, hormonal, and reproductive assessments between the Caucasian and African American women. The African American women had greater average weight, shorter height, greater BMI, an earlier age at menarche, greater parity, and several other clinical/hormonal differences compared to the Caucasian cohort. In both ethnic groups, AFC was most closely associated with serum levels of AMH and FSH, independent of female age (Caucasians: *R* ^2^ = 0.67 and −0.16, respectively; African Americans: *R* ^2^ = 0.49 and −0.14, respectively; *P* < 0.001). AMH levels increased and FSH levels decreased with antral follicle count. While we found that BMI was not associated with the total number of follicles, interestingly BMI was associated with total fraction of African ancestry. Although BMI and follicle counts were higher in the African American cohort this cannot be explained by higher rates of PCOS, as women with PCOS and hyperandrogenism, as well as other reproductive disorders were excluded. It is likely that many of these confounding variables, including ancestral genetic background, may influence the oocyte pool, follicular atresia, and reproductive lifespan, implicating several causative factors in differences between racial groups.

To examine the hypothesis that the ovarian reserve and reproductive lifespan are closely associated and affected by multiple genetic factors, we tested the previously identified variants associated with menopausal age in European women, for association with follicle number, AMH and FSH in our population of women living in the US. We found that variants in the region immediately flanking the top menopausal SNP, rs16991615, were associated with AMH and FSH, in both ethnic groups. Notably, the majority of these variants were linked with AMH, FSH, and/or AFC, and were replicated in both Caucasian and African American women. Together, these results indicate there are genetic associations underlying ovarian reserve and menopausal age (reproductive lifespan) in women of multiple ethnicities. We further found that directions and magnitudes of the effects of all tested menopausal variants were highly similar between age at menopause and follicle number among the Caucasian women—variants associated with later menopausal age were associated with higher follicle counts and vice versa. There were ±0.3 to 1-year differences in menopausal age, which were correlated with ±2 to 3-follicle differences in AFC, providing evidence that these reproductive traits are functionally and genetically related. We found that variant rs16991615 within the *MCM8* gene was significantly associated with later menopausal age of +0.92 to 1.07 years in previous studies (He et al. 2009; Murray et al. 2010; Stolk et al. 2009) and greater follicle numbers of +2.79 ± 1.67 follicles in the Caucasian cohort of this study. This significant non-synonymous SNP in exon 9 of *MCM8* results in an amino acid change from glutamic acid to lysine, which may alter protein function.

*MCM8* is a member of an evolutionarily conserved family of proteins that are involved in whole genome replication. However, MCM8 does not associate with the other MCMs and likely has a role in DNA elongation (after replication licensing). It is expressed in a variety of human cell lines and tissues (Gozuacik et al. 2003) and localizes specifically to the chromatin in the nucleus in S phase of the cell cycle (Gozuacik et al. 2003; Maiorano et al. 2005). Notably, the fly ortholog of *MCM8*, *rec* (*recombination defective*), has a pivotal role in the formation of meiotic crossovers during female gamete development with its loss leading to chromosomal non-disjunction, aneuploidy and diminished fertility (Blanton et al. 2005). *Rec* mutants are specifically unable to copy enough DNA to form linkages between chromosomes during egg meiosis. MCM8 is also expressed in oocytes of the frog, *Xenopus* (Maiorano et al. 2005). Analysis of human EST databases indicates *MCM8* may have moderate expression in both the developing embryo and the ovary (http://www.ncbi.nlm.nih.gov/UniGene), but there are no reports on the expression or role of this highly conserved gene in human gametes.

We discovered that MCM8 is expressed within the human ovary and colocalizes with the germ cell marker, VASA, within the oocytes of several stages of follicle growth. *VASA* is expressed specifically within germ cells and developing oocytes and sperm and is therefore a good marker of human oocytes. MCM8 was also expressed at low levels in peritubular cells, but not in developing sperm within the human testis. Interestingly, in some tissues MCM family members are functionally regulated by estrogen and progesterone (Pan et al. 2006), providing another plausible link between MCMs and ovarian function. Taken together these findings present the first genetic links underlying oocyte number and menopause. There may be direct effects of *MCM8* and other genes on oocyte meiosis, development or depletion, and ultimately, menopausal age providing a mechanism by which they influence the reproductive lifespan. Future in vitro studies can examine the functional roles of *MCM8*, as well as other identified candidate genes in germ cell and follicle development.

If a gene impacts the initial number of oocytes with which a woman is endowed or their rate of depletion, that gene might be expressed in oocytes, follicles and/or the ovary and vary in the human population. Most of the identified genes containing or near by the variants associated with AFC have no previously demonstrated roles in female reproduction, but like *MCM8*, several are expressed in the human ovary. *LRRC61*, proximal to the most significant variant and associated with +7 to 8 follicles, encodes a protein-binding gene and is widely expressed, with highest expression in the human ovary, cervix, and adrenal gland. However, there has been no examination of function in female biology. Other variants are within the *MACROD2* gene at 20p12.1 and nearby the *KLRAP1* gene at 12p13.2. Little is known about the function of *MACROD2*, although it appears to be expressed in several tissues of the body including the ovary and testis. The *TAF4B* gene, nearby an associated variant is expressed in granulosa cells of the follicle in both female mice and women (Freiman et al. 2001; Wu et al. 2005). It is of interest that *TAF4B* null mice are female infertile with defects in folliculogenesis and oocyte maturation and resulting POF (Lovasco et al. 2010) (Ovarian Kaleidoscope database: http://ovary.stanford.edu/). Future functional studies assessing gene expression and genetic silencing and overexpression studies in female cell lines and ovarian tissues are needed to explore the roles of these genes in human oocyte biology.

As the ability of this work to validate many of the variants associated with menopausal age, as well as discover new genome-wide variants associated with follicle number is limited by sample size, future studies may aim for validation of these findings. As this study is the first to perform a GWA on antral follicle number in fertile, normo-ovulatory women and also to examine multiple ethnic groups, there are no other cohorts for independent validation. It is hoped that future work will collect data on AFC to enable cross-validation and multi-site meta-analyses.

In this and previous work we identify a set of candidate genes that are associated with one or more of the human ovarian reserve markers—AFC, AMH, FSH, and menopause (Fig. 5). It is noteworthy that several variants associated with both follicle number and menopause localized to the same genomic regions of 20p12.1–12.3 and 13q (Online Resource Figure 3). Our previous work found common regions on 13q and 12p linked with several ovarian reserve markers (Schuh-Huerta et al. 2012). Further, other work has identified loci on chromosomes 13, 19, and 20 that are associated with age at menopause (Stolk et al. 2009). Therefore, these chromosomal regions and associated genes may be hot spots for causative alleles or genes associated with ovarian reserve and reproductive lifespan. Replication of these findings in independent larger cohorts, as well as longitudinal studies directly measuring follicle number over time and the prospective onset of menopause in this cohort could test the actual phenotypic and genotypic associations between rates of follicle loss and entry into menopause within the same population of women.

In conclusion, we present the first evidence for common variants and new candidate genes underlying follicle number (ovarian reserve), menopause, and the reproductive lifespan. A better understanding of the genes and environmental factors that impact ovarian reserve may provide greater insight on the process of reproductive aging and the genetic requirements of human fertility. Prediction of follicle loss could ultimately enhance reproductive potential and overall health. It is hoped that this work may help form a foundation for clinical applications in identifying women at risk for early ovarian failure and hence associated reproductive and somatic diseases.

## Electronic supplementary material

Below is the link to the electronic supplementary material.

**Supplemental Data** Online Resource material is available at *Hum Genet* online.

Supplementary material 1 (DOC 893 kb)
