# Supplementary material for: Genetic markers of ovarian follicle number and menopause in women of multiple ethnicities
Source: Hum Genet. 2012 Jun 13;131(11):1709–24. doi: 10.1007/s00439-012-1184-0 (PMC3470691; doi:10.1007/s00439-012-1184-0)
Supplement: Supplementary file 1 — Supplementary material 1 (DOC 893 kb) [file 439_2012_1184_MOESM1_ESM.doc]

**ONLINE RESOURCE MATERIAL**

*Schuh-Huerta et al., Genetic markers of ovarian follicle number and menopause in women of multiple ethnicities*

**Subjects and Methods**

Study Population

OVA Study participants were recruited from Kaiser Permanente Northern California (KP), an integrated health care delivery system that provides medical care to over 30% of the population of Northern California. The socio-demographic and health-related characteristics of the KP membership are generally representative of the population of Northern California, as determined by the population-based California Health Interview Study, particularly if the comparison is limited only to those covered by health insurance. OVA participants were similar to the KP population in terms of tobacco use, income, education, and employment status. Women with oligo- or an-ovulation, surgically-diagnosed endometriosis, ovarian failure, polycystic ovary syndrome, or a history of uterine or ovarian surgery were excluded. Women were excluded if they had cysts, fibroids or other abnormalities that obscured AFC measurements. Subjects were also excluded if they had taken oral contraceptive pills (OCPs) or medications containing estrogen or progestin that alter the menstrual cycle within the 3 months prior to enrollment.

Phenotypes and Covariates

A Shimadzu SDU-450XL machine with a variable 4–8 mHz vaginal transducer, was used to take measurements of the transverse, longitudinal, and anteroposterior diameters of each ovary using electronic calipers (supplemental figure S1A). All echo-free structures within the ovaries were regarded as follicles. All follicles with a mean diameter (of 2 dimensions) of 2–10 mm were counted (figure 1A, supplemental figure S1B). The total AFC for each woman was determined by summing the AFCs for both ovaries. To eliminate experimental variability all women were examined at the same time in their menstrual cycle, using the same equipment, and women with unclear ultrasounds were excluded. Ultrasound examinations were performed by one of two experienced physicians; concordance between them is at 95% with a strong correlation between repeated measurements (R^2^ = 0.92).

The age of each woman at the time of the AFC measurement was rounded off to the nearest 10^th^ of a year. Body measurements including weight, height, and waist and hip circumference, were also obtained. BMI (height/weight^2^) was calculated for each woman, as well as waist-hip and waist-height ratios. Age at menarche was retrospectively ascertained by recall in questionnaires and reported as the age in whole years. Average cycle length in days over the last 12 months was determined retrospectively and the last 2 cycles were recorded prospectively. Parity or the number of children born to each woman was assessed. Cigarette smoke exposure and history of taking oral OCPs were also quantified and analyzed.

Genotyping

The Genome-Wide Human SNP Array 6.0 was used for genotyping. This array contains more than 900,000 SNP markers and 940,000 copy number variant (CNV) markers; approximately 1 marker every 700 bases across the genome. Stringent quality control, concordance checks, and SNP filtering were employed using Genotyping Console (GTC) v.4.0 software (http://www.affymetrix.com) to eliminate poor samples and poorly performing or rare SNPs. The quality control (QC) measures used were: QC Call Rate or percentage of a specific set of control SNPs genotyped or ‘called’ in the samples using the Dynamic algorithm (filtered for QC Call Rate ≥ 86%); and Contrast QC (filtered for Contrast QC ≥ 0.4). Contrast QC, the most important QC measure, uses a static set of 10,000 randomly chosen SNPs and quantifies the difference between the highest and lowest contrast values of the homozygote and heterozygote genotypes. Laboratory technical quality control criteria also included SNP fingerprints for sample tracking and early detection of sample misidentification, the use of HapMap controls to check genotype quality, gender confirmation based on mean intensities of SNP probes on the X and Y chromosomes, and tracking of reagent and instrument performance.

GTC 4.0 was used for genotyping. It uses the Birdseed algorithm and performs a multiple-chip analysis to estimate signal intensity for each allele of each SNP, fitting probe-specific effects to increase precision. Clustering analysis and examination of relative sample intensity, as well as filtering for high sample call rates (≥ 95%) ensured high sample quality prior to downstream association analysis. Individual SNPs across the genome were filtered for SNP call rates > 95%, missing call rate (MCR) < 5%, minor allele frequency (MAF) > 1%, and lack of significant deviation from Hardy-Weinberg Equilibrium (HWE; *P* > 0.007). Of 909,622 SNP probes on the array, 677,261 and 738,185 in the Caucasian and African American cohorts, respectively, passed QC and SNP filter criteria and were independently tested for association with AFC.

Population Structure

PCA was carried out within R v2.11.1 and BEAGLE v3.0.2. Briefly, genotypes at 10,000 SNPs across the genome with low levels of linkage disequilibrium and minor allele frequency (MAF) > 0.01, were coded as 0, 1 or 2 (AA, AB, BB) and were mean-centered. The OVA cohorts were combined with the HapMap CEU and YRI cohorts, missing genotypes were imputed using the program BEAGLE v.3.0.2, and outliers were identified. Fraction of African ancestry was also analyzed among subjects and allele frequency spectra were compared between the Caucasian, CEU, African American and YRI cohorts to analyze allele composition and population homogeneity.

SNP Association Analysis

The allelic test of the Fisher’s exact test determined associations with follicle number using the programs BEAGLE v.3.0.2 and quantreg v.4.44, within R v.2.11.1. The correlation coefficients and effect sizes (the difference in the mean number of follicles ± SEM from the regression fit) were then determined by regression analyses for a given allele or genotype. Independent analyses were performed on AFC as either a quantitative or binary trait. In the binary analysis, using the application quantreg, AFC verses age was first separated by a robust or quantile regression into “high” or “low” AFC (similar to case vs. control association studies); high AFCs were those above and low AFCs were those below, the robust regression fit. AFC was analyzed as a binary trait due to its clinical relevance and the categorization of women with high, low (POF risk) or normal AFC for their given age. For the association analysis with AFC, Caucasian women over age 43 were excluded due to low sample sizes, low follicle numbers, and decreased variability at these upper ages. Due to the somewhat arbitrary nature of the clinical high/low AFC categorization we used a second method as a form of validation. In parallel analyses, allelic and genotypic tests using the Kruskal Wallace Rank-Sum Test determined SNPs significantly associated with AFC, as a quantitative variable. For each SNP allele and genotype, a linear regression of AFC verses age was obtained, the residual was computed for each woman, and the women were ranked into groups based on the allele/genotype to determine whether one allele/genotype had higher or lower residuals (effect sizes) than expected by random chance alone. It is of note that the Fisher’s exact test and the Kruskal Wallace Test, as well as analyzing AFC as a categorical and quantitative variable, resulted in highly similar associations, effect sizes, and the same top hits.

Linkage Disequilibrium (LD) encompassing associated SNPs, the SNPs involved in the signal, recombination rates, and associated genes were analyzed within BEAGLE v.3.0.2, Haploview v.4.2 (http://www.broadinstitute.org/mpg/haploview) and LocusZoom (http://csg.sph.umich.edu/locuszoom/). LD tagging was performed with the Tagger program within Haploview v.4.2, and similar methods within LocusZoom, to capture SNPs with squared correlation coefficients of r^2^ > 0.8.

To estimate the power of the study to identify associations with AFC we performed power calculations incorporating our sample size (~250 women), the effect size (multiplicative effect of each minor allele), MAF (of 0.1, 0.2, 0.3, and 0.5), and estimates of noise in our samples (using Poisson Distribution). There was approximately 60% power to detect SNPs with an MAF of 0.5 and an effect size of 0.4 (40% difference) per minor allele at *P* < 0.05. We had 80% power to detect an association with an MAF of 0.5 and an effect size of ~0.45 or an MAF of ≥ 0.3 and an effect size of ~0.8. At an MAF of 0.2 we had about 20% power. Due to our sample size therefore, we had the greatest power to detect associations that had relatively high MAFs and large effects on AFC. Final *P* values were computed and analyzed at both the chromosome- and genome-wide-levels, due to our sample size/power constraints. Further, as several chromosomes have loci potentially associated with ovarian function (ie. Chr X, 20, 12, 13), variants were also analyzed specifically at the chromosome level.

**Results**

Description of Population Phenotypes and Covariates

In the Caucasian cohort, the average height and weight were 166.4 cm (5’6”) and 67.7 kg (149 lbs), respectively, with an average body mass index (BMI) of 24.4 ± 0.4. In the African American cohort, the average height and weight were 164.3 cm (5’5”) and 87.0 kg (192 lbs), respectively, with an average BMI of 32.1 ± 0.6. There were significant differences between the body measurements of Caucasian and African American women. The African American cohort had significantly shorter height (*P* = 0.00035), greater weight (*P* = 2.54 x 10^-21^), higher waist-hip (*P* = 1.02 x 10^-18^) and waist-height (*P* = 2.47 x 10^-31^) ratios, and significantly greater BMI (*P* = 1.58 x 10^-27^). More than 52% of the African American women fell within the obese category (106 women with BMI > 30).

Age at menarche differed between Caucasians and African Americans at 12.8 ± 0.09 vs. 12.1 ± 0.1 years (range = 9–17 years; *P* = 5.87 x 10^-6^). Menstrual cycle length was similar between Caucasian and African American women at 29.1 ± 0.1 and 29.6 ± 0.2 days, respectively, with greater than 70% of the women having cycle lengths of 28–32 days. A significant proportion of the women had demonstrated fertility with 36% and 52% of the Caucasian and African American women, respectively, having a parity of one or more children, and the remainder of the women having no indications of reproductive disorders or infertility.

Smoking and OCP use were quantified similar to our previously published work. Controlling for smoking and OCP use in the genome-wide analysis did not affect the associations, indicating the effects of the variants likely occur directly on AFC/ovarian reserve, rather than through these covariates.


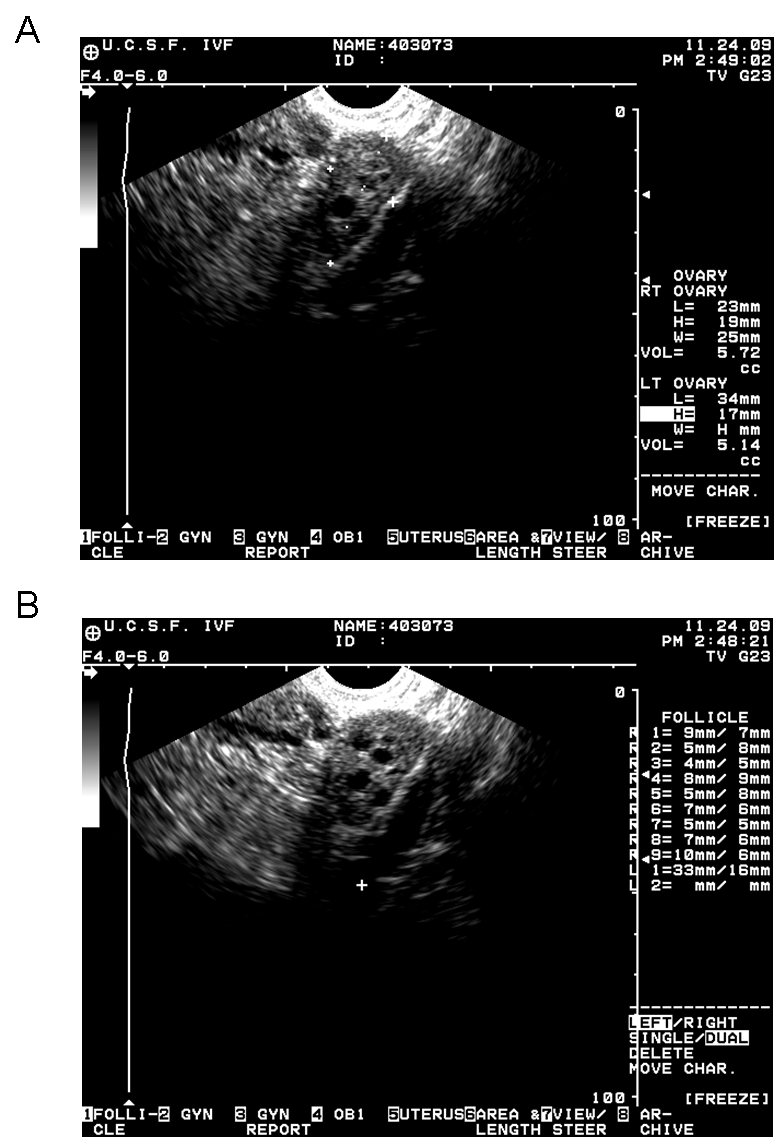
**Figure 1**

**Figure 1.** Transvaginal ultrasound examination of antral follicle number: (**A**) Shown is an ultrasound image of the left ovary of one woman of the study, taken while obtaining length, height, width, and volume measurements of the ovary; (**B**) An ultrasound image from the same woman, taken during the antral follicle count procedure. This woman had several antral follicles between 1–10 mm.

**Figure 2**

**Figure 2.** Ethnicity validation and tests of population stratification in Caucasian and African American cohorts: (**A**) PCA with singular value decomposition on 10,000 SNPs across the genome indicates the Caucasian OVA cohort (blue points) clusters with the HapMap CEU population (black points) and the African American OVA cohort (yellow points) clusters near the HapMap YRI population (red points). In all analyses the same three outliers were identified (enclosed in dashed circles), which clustered near or within the alternate ethnic population. (**B**) In additional PCA tests, fraction of African Ancestry was analyzed and allele frequency spectra were compared between the Caucasian and CEU, and African American and YRI/CEU cohorts. The Caucasian cohort displayed an allele frequency spectrum nearly identical to the CEU population, while the African American cohort displayed an allele frequency spectrum closely correlated with a combination of 80% YRI and 20% CEU alleles, which matches that of individuals of African ancestry living in America.


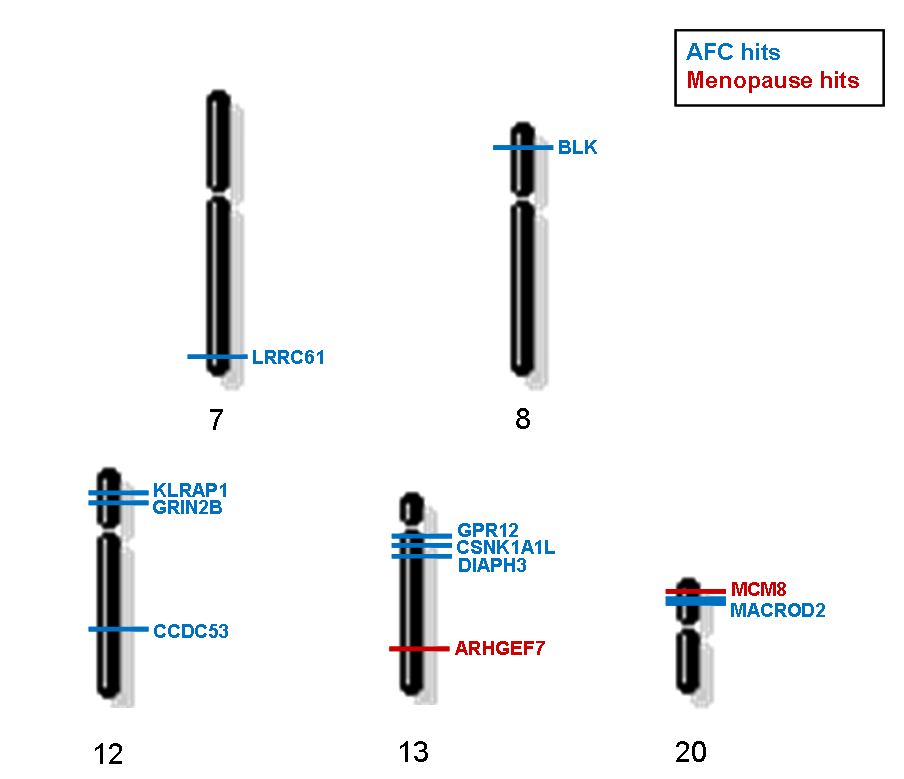
**Figure 3**

**Figure 3.** Genomic regions linked with follicle number and menopause. Shown are chromosomes 7, 8, 12, 13, and 20 with the locations and genes associated with variants linked with menopause (red) and/or follicle number (blue) in Caucasian or African American women. On chromosome 20, rs16991615, associated with both menopause and follicle number is about 9 MB away from rs175810, which is associated with follicle number. On the long arm of chromosome 13, 4 variants were associated with either menopause or antral follicle count – rs7333181 (menopause in Europeans); rs4769524 (follicle number in Caucasians); rs17191595 (follicle number in African Americans); and rs17055644 (follicle number in Caucasians and African Americans). Genes: *KLRAP1*, *Killer Cell Lectin-Like Receptor Subfamily A, Pseudogene Member 1;* *GRIN2B*, *Glutamate Receptor, Ionotropic, N-Methyl D-Aspartate 2B; CCDC53, Coiled-Coil Domain Containing 53; GRP12, G Protein-Coupled Receptor 12; CSNK1A1L, Casein Kinase 1, α 1-Like; ARHGEF7, Rho Guanine Nucleotide Exchange Factor 7; MCM8, Minichromosome Maintenance Complex Component 8; MACROD2, MACRO Domain Containing 2.*

**Supplemental Tables**

**Table 1. Genotyping results of Caucasian and African American cohorts**

|  | **Caucasian** |  | **African Amer** |  |
| --- | --- | --- | --- | --- |
|  | **n** | **Mean ± SEM** | **n** | **Mean ± SEM** |
| Subjects Enrolled | 273 |  | 245 |  |
| Subjects Genotyped | 249 |  | 203 |  |
| Subjects Excluded | 24 |  | 28 |  |
| Anatomical/ovarian problems | 21 |  | 27 |  |
| Failed Quality Control | 1 |  | 0 |  |
| Failed Ethnicity Validation | 2 |  | 1 |  |
| Subjects Included in Analysis | 245 |  | 202 |  |
| Genotyping Quality Control (QC)**^a^** |  |  |  |  |
| Contrast Quality Control (QC) | 245 | 1.93 ± 0.046 | 202 | 2.22 ± 0.035 |
| QC Call Rate (%) | 245 | 94.0 ± 0.2 |  | 97.0 ± 0.12 |
| Sample Call Rates (%) | 245 | 98.0 ± 0.07 |  | 99.4 ± 0.08 |
| SNP Markers Filtered | 909,622 |  |  | 909,622 |
| SNPs Analyzed for Association**^b^** | 677,261 |  |  | 738,185 |

^a^ Filtered for: Contrast QC > 0.4, QC Call Rates > 86%, and Sample Call Rates > 95%

^b^ Filtered for: SNP call rates > 95%, minor allele frequency > 0.01, and lack of deviation from Hardy-Weinberg

Equilibrium (*P* > 0.007)

**Table 2.** Lack of associations between known menopause-related SNPs and follicle number in African American women of OVA Study

| **SNP^a^** | **Gene** | **Cytoband** | **Alleles^b^** | **MAF** | **Menopause GWAS Genotype Effect (years)^c^** | **OVA Study Genotype Effect (follicles)^d^** | ***P* value^e^** |
| --- | --- | --- | --- | --- | --- | --- | --- |
|  |  |  |  |  |  |  |  |
| rs16991615 rs17835738 | *MCM8* | 20p12.3 | A/G | 0.01 | +1.07 ± 0.11 | -2.31 ± 5.16 | 0.094 (0.38) |
| rs4806660 | *TMEM150B* | 19q13.42 | G/A | 0.37 | -0.41 ± 0.030 | +3.48 ± 2.18 | 0.089 (0.36) |
| rs691141 | *HK3* | 5q35.2 | A/G | 0.43 | +0.36 ± 0.052 | +1.96 ± 1.69 | 0.23 (0.92) |
| rs2153157 | *SYCP2L* | 6p24.2 | A/G | 0.30 | +0.29 ± 0.052 | +1.86 ± 0.97 | 0.91 (1) |

^a^ SNPs at each locus are those published for association with menopausal age, rather than those with the strongest signal for follicle count

^b^ Shown as minor/major allele

^c^ The difference in mean (± SEM) age at menopause in years per copy of the SNP minor allele as previously reported

^d^ The difference in mean (± SEM) number of follicles for the corresponding genotype calculated from the regression analysis.

^e^ *P-* values are based on the Fisher’s exact test for SNP association with antral follicle count; parentheses denote corrected *P* values.

**Table 3.** Validation of ovarian reserve markers: Associations between antral

follicle count-related SNPs and AMH levels in Caucasian women of OVA Study

| **SNP^a^** | **Chr** | **Gene** | **Alleles^b^** | **AFC *P*- value rank^c^** | **AMH Level**  ***P*-value^d^** | **Corrected**  ***P*-value^e^** |
| --- | --- | --- | --- | --- | --- | --- |
|  |  | |  |  |  |  |
| rs4769524 | 13q12.13 | ***GPR12*** | A/G | 6 | 7.65 x 10^-4^ | ***0.01224*** |
| rs7329984 | 13q12.13 | ***GPR12*** | G/A | 10 | 1.47 x 10^-3^ | ***0.023552*** |
| rs2417903 | 12p13.2 | ***KLRAP1*** | A/T | 2 | 2.04 x 10^-3^ | ***0.032576*** |
| rs7305642 | 12p13.2 | ***KLRAP1*** | G/A | 3 | 2.04 x 10^-3^ | ***0.032576*** |
| rs175810 | 20p12.1 | ***MACROD2*** | G/C | 4 | 2.56 x 10^-3^ | ***0.040912*** |
| rs1382566 | 8p23.1 | ***BLK*** | C/G | 5 | 3.12 x 10^-3^ | ***0.049904*** |
| rs761684 | 20p12.1 | ***MACROD2*** | T/C | 11 | 3.20 x 10^-3^ | ***0.0512*** |
| rs6488306 | 12p13.2 | *KLRC1 & KLRAP1* | C/T | 16 | 3.49 x 10^-3^ | 0.055872 |
| rs12485985 | 3p22.3 | *TRIM71* | G/A | 8 | 8.46 x 10^-3^ | 0.135376 |
| rs9845475 | 3p22.3 | *TRIM71* | C/A | 12 | 1.25 x 10^-2^ | 0.20016 |
| rs11698484 | 20p12.1 | *PCSK2* | T/C | 13 | 1.87 x 10^-2^ | 0.2992 |
| rs17835387 | 1q42.2 | *SIPA1L2* | G/A | 15 | 2.62 x 10^-2^ | 0.41968 |
| rs6043560 | 20p12.1 | *MACROD2* | T/C | 14 | 3.26 x 10^-2^ | 0.52192 |
| rs17835738 | 7q36.1 | *LRRC61* | G/C | 1 | 7.79 x 10^-2^ | 1 |
| rs739826 | 7p12.1 | *COBL* | A/T | 7 | 3.07 x 10^-1^ | 1 |
| rs246240 | 16p13.11 | *ABCC1* | G/A | 9 | 6.44 x 10^-1^ | 1 |

*P* ≤ 0.05 for 7 out of 16 AFC-associated SNPs also associated with AMH levels, compared to the 1.45 expected by chance.

^a^ SNPs at each locus are those associated with antral follicle count, rather than those with the strongest signal for AMH.

^b^ Shown as minor/major allele

^c^ The rank of each SNP associated with follicle count in terms of lowest *P*-values (most significant hits)

^d^ *P*-values are based on the Fisher’s exact test for association with AMH levels (pM)

^e^ *P*-values corrected for the number of tests by Bonferroni correction (significant *P*-values in bold).
